# Comparative Outcomes of Rotational Versus Orbital Atherectomy in Calcified Coronary Lesions: An Updated Systematic Review and Meta‐Analysis

**DOI:** 10.1155/cdr/4716803

**Published:** 2026-09-02

**Authors:** Eyad Jamileh, Ahmed Elmewafy, Fahad Muhammad, Ahmed Ali Kayyale, Usama Al Bushe Ade, Abdelmadjid Zinet, Umar Faruq Ali, Ibrahim Antoun

**Affiliations:** ^1^ East Lancashire Hospitals NHS Trust, Blackburn, England, UK, nhs.uk; ^2^ School of Medicine, Queen′s University Belfast, Belfast, Northern Ireland, UK, qub.ac.uk; ^3^ School of Medicine, University of Sheffield, Sheffield, England, UK, sheffield.ac.uk; ^4^ School of Medicine, Queen Mary University of London, London, England, UK, qmul.ac.uk; ^5^ Princess Alexandra Hospital NHS Foundation Trust, Harlow, England, UK; ^6^ Sandwell and West Birmingham NHS Trust, Birmingham, England, UK, nhs.uk; ^7^ Imperial College Healthcare NHS Trust, London, England, UK, nhs.uk; ^8^ Division of Cardiovascular Sciences, Clinical Science Wing, University of Leicester, Glenfield Hospital, Leicester, England, UK, nhs.uk

**Keywords:** calcified coronary lesions, coronary artery disease, orbital atherectomy, percutaneous coronary intervention, rotational atherectomy

## Abstract

**Introduction:**

Severe coronary artery calcification (CAC) can complicate percutaneous coronary intervention (PCI) and is associated with less favourable procedural and clinical outcomes. Rotational atherectomy (RA) and orbital atherectomy (OA) are established calcium‐modification strategies used to facilitate PCI in heavily calcified lesions. This systematic review and meta‐analysis evaluated the comparative clinical and procedural outcomes of RA and OA in patients with severe CAC.

**Methods:**

This systematic review and meta‐analysis was conducted in accordance with PRISMA 2020 guidance. PubMed, EMBASE, and CENTRAL were searched from inception to July 2026 for studies directly comparing RA and OA in patients undergoing PCI for severe CAC. Eligible studies were synthesised quantitatively where appropriate and narratively otherwise. Risk of bias was evaluated using the Newcastle–Ottawa Scale for observational studies and the Cochrane Risk of Bias 2 tool for the randomised controlled trial.

**Results:**

No statistically significant differences between RA and OA were identified for short‐term major adverse cardiovascular events (MACE), short‐ or long‐term all‐cause mortality, cardiac mortality, long‐term nonfatal myocardial infarction (MI) or short‐term target vessel revascularisation (TVR). Compared with OA, RA was associated with higher odds of long‐term MACE (OR 1.51, 95% CI 1.01–2.26), short‐term nonfatal MI (OR 1.77, 95% CI 1.05–2.98) and long‐term TVR (OR 2.51, 95% CI 1.12–5.64). Conversely, coronary dissection occurred less frequently with RA (OR 0.38, 95% CI 0.20–0.72). There were no significant between‐group differences in cardiac tamponade, device‐induced coronary perforation, slow‐flow/no‐reflow, fluoroscopy time or contrast volume.

**Conclusion:**

In this updated meta‐analysis of more than 80,000 patients, OA was associated with lower pooled rates of long‐term MACE, short‐term nonfatal MI and long‐term TVR, whereas RA was associated with a lower rate of coronary dissection. No consistent difference in all‐cause mortality was demonstrated. Given the predominantly observational evidence base, these associations should not be interpreted as evidence of causal superiority, and device selection should remain individualised according to lesion characteristics, intravascular imaging findings and operator expertise.

## 1. Introduction

Coronary artery calcification (CAC) is commonly encountered during percutaneous coronary intervention (PCI) and is present in a substantial proportion of patients undergoing coronary revascularisation. Increasing calcific burden is associated with greater procedural difficulty and may adversely affect both immediate and subsequent cardiovascular outcomes [1, 2]. Although severe CAC may be quantified noninvasively using computed tomography, with an Agatston score of ≥ 400 frequently regarded as indicative of extensive calcification, lesion severity during PCI is more commonly characterised using angiographic or intravascular imaging criteria. Definitions of severe calcification therefore differ between clinical studies [3].

Adequate modification of heavily calcified plaque is often required before stent implantation because rigid calcium can impair device delivery, limit stent expansion and compromise the final procedural result. Rotational atherectomy (RA) and orbital atherectomy (OA) represent two established approaches to plaque modification in this setting. RA employs a rapidly rotating diamond‐coated burr, typically operating at approximately 140,000–190,000 revolutions per minute, to preferentially ablate relatively inelastic calcified tissue through differential cutting [4]. OA differs mechanically by using an eccentrically mounted diamond‐coated crown that moves in an orbital trajectory within the vessel and can operate at speeds of up to approximately 120,000 revolutions per minute [5]. The resulting circumferential sanding action and bidirectional movement may permit treatment of a wider portion of the calcified vessel wall [6]. Despite these mechanistic differences, both techniques are intended to improve lesion preparation and facilitate satisfactory stent deployment in complex calcified PCI.

Previous meta‐analyses comparing the two approaches have generally suggested broadly similar rates of major adverse cardiovascular events (MACE), although potential differences in individual procedural and clinical outcomes have been reported. Earlier evidence suggested that OA may be associated with shorter fluoroscopy times and a lower incidence of short‐term MI while potentially carrying a greater risk of coronary perforation [7]. Interpretation of these findings, however, has been constrained by relatively small study populations, dependence on nonrandomised evidence, variable follow‐up periods and limited representation of contemporary intravascular imaging‐guided PCI.

Since these earlier syntheses were undertaken, further registry data and randomised evidence comparing RA and OA have become available, whereas atherectomy practice and imaging‐guided lesion assessment have continued to evolve [8, 9]. Reassessment of the comparative evidence is therefore appropriate. The present systematic review and meta‐analysis aimed to provide an updated comparison of RA and OA in severe CAC, with separate evaluation of short‐ and long‐term clinical outcomes as well as procedural complications and characteristics.

## 2. Methods

This systematic review and meta‐analysis was conducted and reported in accordance with the Preferred Reporting Items for Systematic Reviews and Meta‐Analyses (PRISMA) 2020 statement [10]. The protocol was prospectively registered in the International Prospective Register of Systematic Reviews (PROSPERO; CRD420251113632) [11]. The completed PRISMA 2020 checklist is provided in File S1.

### 2.1. Eligibility Criteria

Eligibility criteria were specified prospectively in the registered protocol [11]. Studies were considered eligible when they directly compared RA and OA in patients undergoing PCI for severely calcified coronary lesions. Because the definition of severe CAC was not uniform across the available literature, the criteria applied by the investigators of each original study were accepted. These definitions could be based on computed tomography, coronary angiography or intravascular imaging.

### 2.2. Primary Outcomes

The principal outcome was MACE, using the definition reported by each included study. As individual studies differed in the components incorporated within their composite MACE endpoint, the original study‐level definition was retained for analysis. Individual clinical outcomes of interest were all‐cause mortality, cardiac mortality, nonfatal myocardial infarction (MI) and target vessel revascularisation (TVR).

### 2.3. Secondary Outcomes

Secondary endpoints comprised procedural complications and procedural characteristics. Complications of interest included intraprocedural coronary dissection, cardiac tamponade, slow‐flow/no‐reflow and device‐related coronary perforation. Procedural measures included fluoroscopy duration and contrast volume.

### 2.4. Literature Search Strategy

PubMed, EMBASE and the Cochrane Central Register of Controlled Trials (CENTRAL) were systematically searched from database inception to July 2026. Full database‐specific search strategies are provided in File S2. Searches for additional ongoing, unpublished or grey‐literature studies were undertaken using the World Health Organization International Clinical Trials Registry Platform, ClinicalTrials.gov and the ISRCTN Registry. Reference lists of eligible studies and relevant systematic reviews were also manually examined to identify further potentially eligible reports.

### 2.5. Study Selection

Two reviewers (E.J. and A.E.) independently screened retrieved records at title and abstract level without access to each other′s decisions. Articles considered potentially eligible underwent full‐text assessment. Disagreements regarding inclusion were resolved by discussion, with consultation of a third reviewer (I.A.) when consensus could not initially be reached.

### 2.6. Data Extraction and Management

A standardised Microsoft Excel extraction form based on the Cochrane data collection framework for intervention reviews was developed and piloted before formal data collection. Two reviewers independently extracted the required information, and discrepancies were reconciled through discussion. Extracted variables included study‐level characteristics such as author, publication year, country, study design and sample size; participant characteristics including age, sex and relevant clinical background; procedural information relating to RA, OA and procedural guidance; and data for all prespecified primary and secondary outcomes.

### 2.7. DData Synthesis

Quantitative pooling was undertaken when an outcome was reported by at least two eligible studies. Dichotomous outcomes were summarised using odds ratios (ORs), whereas continuous variables were analysed using mean differences (MDs). Random‐effects models were applied throughout. Analyses were performed using Review Manager Version 5.3 (RevMan), and pooled estimates were reported with 95% confidence intervals (CIs).

Statistical heterogeneity was examined using Cochran′s Q test and quantified using the *I* ^2^ statistic. *I* ^2^ values of 0%–25%, 25%–75% and 75%–100% were interpreted as indicating low, moderate and high heterogeneity, respectively [12].

### 2.8. SSensitivity Analysis

A prespecified sensitivity analysis evaluated the influence of Megaly et al. [13], which contained a substantially larger patient population than the remaining included studies and demonstrated considerable imbalance between the treatment cohorts. Analyses excluding this study were used to assess whether pooled estimates remained stable when its statistical influence was removed.

### 2.9. Risk of Bias and Quality Assessment

Two reviewers independently evaluated methodological quality. The randomised controlled trial was assessed using the Cochrane Risk of Bias 2 (RoB 2) tool. Observational studies were evaluated using the Newcastle–Ottawa Scale (NOS), which considers study selection, comparability between groups and adequacy of outcome ascertainment and follow‐up.

**Figure 1:**
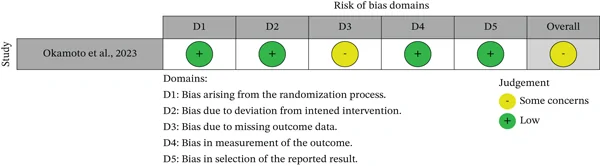
Risk of bias assessment for the randomised controlled trial included in the meta‐analysis, evaluated using the Cochrane Risk of Bias 2.0 tool.

Observational studies received a maximum possible score of nine stars, with higher scores reflecting greater methodological quality. The Agency for Healthcare Research and Quality (AHRQ) criteria were additionally used to categorise studies as good, fair or poor quality. Risk‐of‐bias findings are presented in Table 1 and Figure 1.

**Table 1:** Risk of bias assessment for the observational studies included in the meta‐analysis, evaluated using the Newcastle–Ottawa Scale (NOS).

Author (year)	Selection (out of 4 stars)	Comparability (out of 2 stars)	Outcome (out of 3 stars)	Quality assessment based on the Agency for Healthcare Research and Quality (AHRQ)
Yasumura et al. (2024) [14]	∗∗∗∗	∗∗	∗∗∗	Good
Rola et al. (2023) [15]	∗∗∗∗	**—**	∗∗∗	Poor
Megaly et al. (2021) [13]	∗∗∗∗	∗∗	∗∗∗	Good
Barrett et al. (2020) [16]	∗∗∗	∗∗	∗∗∗	Good
El Hajj et al. (2020) [17]	∗∗∗	∗	∗∗	Good
Okomoto et al. (2019) [18]	∗∗∗∗	∗∗	∗∗	Good
Chambers et al. (2018) [19]	∗∗∗∗	∗	∗∗∗	Good
Koifman et al. (2018) [20]	∗∗∗∗	**—**	∗∗∗	Poor
Meraj et al. (2018) [21]	∗∗∗∗	∗∗	∗∗∗	Good
Lee et al. (2017) [22]	∗∗∗∗	∗∗	∗∗∗	Good

Assessment of small‐study effects using funnel plots and Egger′s regression was planned only for outcomes represented by at least 10 studies. Formal testing was not undertaken when fewer than 10 studies contributed to an outcome because methods for detecting funnel‐plot asymmetry have limited reliability with small numbers of studies. Formal GRADE assessment of certainty was not undertaken because the evidence base was predominantly composed of retrospective observational studies, for which the starting certainty for the principal comparative outcomes would be low.

## 3. Results

### 3.1. Literature Search Results

**Figure 2:**
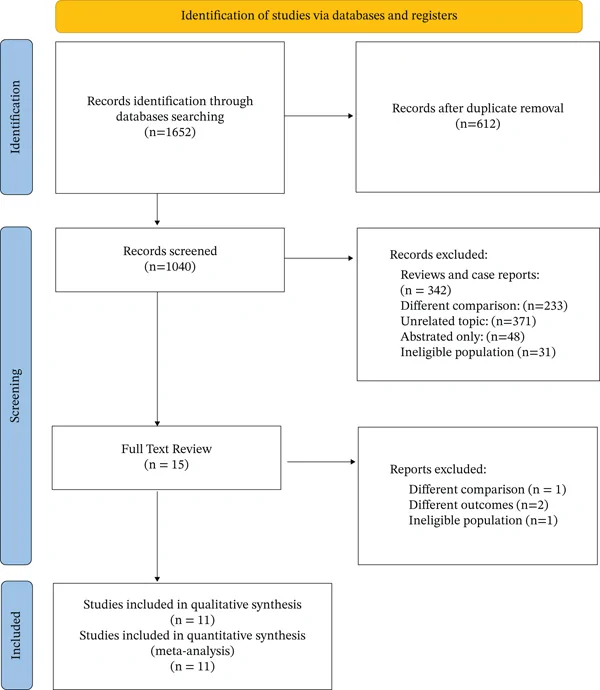
PRISMA chart.

Our search strategy retrieved 1654 studies. After screening the retrieved articles, the authors identified 11 studies that met the eligibility criteria: 10 observational retrospective studies and 1 randomised controlled trial (Figure 2).

### 3.2. Description of the Studies

**Figure 3:**
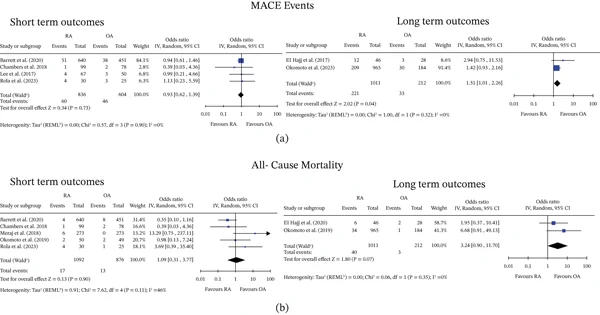
(a) Forest plot of RA versus OA number of MACE events (*k* = 4 for short‐term and *k* = 2 for long‐term); (b) forest plot of RA versus OA number of all‐cause mortality events (*k* = 5 for short‐term and *k* = 2 for long‐term).

**Figure 4:**
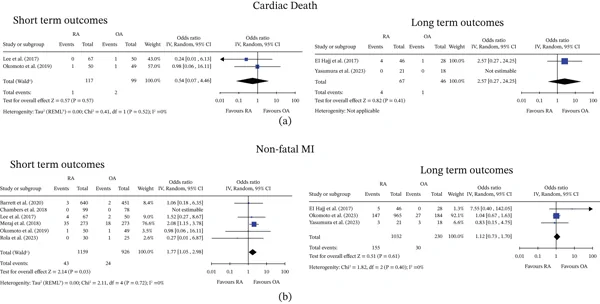
(a) Forest plot of RA versus OA number of cardiac death events (*k* = 2 for short‐term and *k* = 2 for long‐term); (b) forest plot of RA versus OA number of nonfatal MI events (*k* = 6 for short‐term and *k* = 3 for long‐term).

**Figure 5:**
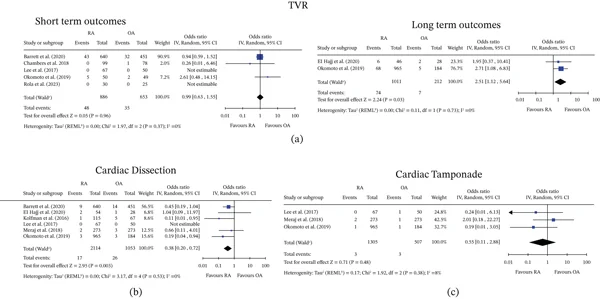
(a) Forest plot of RA versus OA number of TVR events (*k* = 5 for short‐term and *k* = 2 for long‐term); (b) forest plot of RA versus OA number of coronary dissection events (*k* = 6); (c) forest plot of RA versus OA number of cardiac tamponade events (*k* = 3).

**Figure 6:**
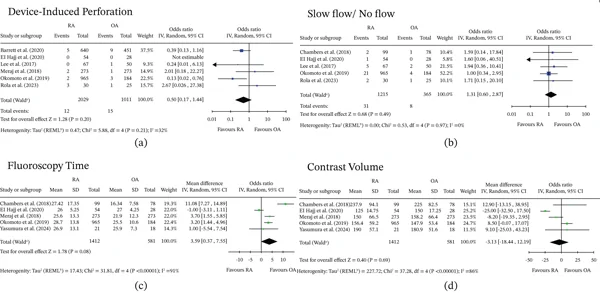
(a) Forest plot of RA versus OA number of device‐induced perforation events (*k* = 6); (b) forest plot of RA versus OA number of slow flow/no‐flow events (*k* = 5); (c) forest plot of RA versus OA mean fluoroscopy time (*k* = 5); (d) forest plot of RA versus OA mean contrast volume used (*k* = 5).

A total of 11 studies were appraised, with a total of 80,924 patients [13–23]. A total of 74,119 patients were included in the RA group and 6805 in the OA group. Tables 2 and 3 summarise the characteristics of the patients included in the studies. Forest plots for each outcome are displayed in Figures 3, 4, 5 and 6.

**Table 2:** Baseline characteristics of patients of individual studies.

**Variables**	**Okamoto et al. 2023 [** **23** **]**		**Yasumura et al. 2024 [** **14** **]**		**Rola et al. 2023. [** **15** **]**		**Barrett et al. 2020 [** **16** **]**	
	**N** = 100		**N** = 39		**N** = 55		**N** = 1091	
	**RA**	**OA**	**RA**	**OA**	**RA**	**OA**	**RA**	**OA**

Number of patients	50	50	21	18	30	25	640	451
Age, years	73.4 ± 9.9	74.2 ± 8.7	66.8 ± 10.0	65.9 ± 6.6	70.4 ± 8.9	68.4 ± 7.9	69.8 ± 8.0	70.9 ± 8.1
Male sex %	72	67	81	83	70	68	98.8	98.7
BMI	24.2 ± 4.2	23.6 ± 4.1	27.6 ± 3.7	30.6 ± 5.6	—	—	—	—
Medical history %								
MI	28	22	—	—	47	48	47	47.5
PCI	44	48	67	61	53	44	—	—
DM	62	60	33	67	53	44	—	—
Smoke	6	8	14	6	—	—	73.6	67.2
HTN	80	88	19	89	87	96	—	—
Stroke	—	—	—	—	—	—	—	—
HLD	58	76	90	93	70	96	—	—
Previous CABG	4	4	0	0	20	8	—	—
LVEF	66 ± 9.3	65 ± 10.1	53.1 ± 9.9	59.0 ± 8.5	—	—	—	—
PAD	—	—	5	11	—	—	30.8	37.3
CKD/dialysis	—	—	14	11	43	24	32.5	31.7
Presentation %								
Stable angina	—	—	90	78	43	24	—	—
UA	—	—	10	22	23	12	—	—
NSTEMI	—	—	0	0	30	64	—	—
STEMI	—	—	0	0	3	0	—	—

**Variables**	**Lee et al. 2017 [** **22** **]**		**Meraj et al. 2018 [** **21** **]**		**Koifman et al. 2018 [** **20** **]**		**El Hajj et al. 2020 [** **17** **]**	
	**N** = 117		**N** = 907		**N** = 184		**N** = 75	
	**RA**	**OA**	**RA**	**OA**	**RA**	**OA**	**RA**	**OA**

Number of patients	67	50	474	433	117	67	46	24^a^
Age, years	61 ± 12	62 ± 11	73.5 ± 9.9	71.0 ± 11.2	74 ± 10	73 ± 11	70.7 ± 7.1	70.1 ± 6.8
Male sex %	69	68	66.5	64	66	72	100	100
BMI	—	—	28.1 ± 5.8	28.3 ± 5.8	30 ± 7	28 ± 6	30 0.3 ± 5.0	30.5 ± 5.1
Medical history %								
MI	24	22	35.2	34.9	30	31	48	21
PCI	28	30	47.3	44.8	45	29	54	25
DM	39	36	53.2	51.7	56	45	65	86
Smoke	—	—	9.7	19.2	44	40	20	38
HTN	66	76	95.8	95.1	98	92	98	86
Stroke	7	6	19.6	15.5	—	—	—	—
HLD	64	72	91.8	92.4	96	85	93	93
Previous CABG	12	10	24.5	14.1	30	22	26	14
LVEF	52 ± 13.1	51 ± 12.7	—	—	50 ± 15	48 ± 16	—	—
PAD	—	—	19.8	17.5	25	27	26	21
CKD/dialysis	—	—	7	7.2	24	21	—	—
Presentation %								
Stable angina	—	—	22.4	19.9	—	—	13	7
UA	22	24	58.4	61.9	—	—	44	57
NSTEMI	10	8	11.2	15	—	—	15	11
STEMI	0	0	1	0.5	—	—	4	0

**Variables**	**Megaly et al. 2021 [** **13** **]**		**Chambers et al. 2018 [** **19** **]**		**Okamoto et al. 2019 [** **18** **]**			
	**N** = 77,040		**N** = 177		**N** = 1149			
	**RA**	**OA**	**RA**	**OA**	**RA**	**OA**		

Number of patients	71,610	5430	99	78	965	184		
Age, years	64 ± 8.5	73 ± 10.2	71.6 ± 8.3	69.5 ± 8.9	71.1 ± 10.3	71.0 ± 10.9		
Male sex %	71	—	71.8	78.8	71.4	74.5		
BMI	—	—	—	—	28.1 ± 5.7	—		
Medical history %								
MI	17	18	25.6	45.5	26.3	20.1		
PCI	2	2	69.2	60.6	—	—		
DM	36	49	51.3	36.4	48.9	39.1		
Smoke	—	—	69.2	87.9	—	—		
HTN	57	67	92.3	93.9	94.1	91.8		
Stroke	6	9	—	—	—	—		
HLD	—	—	84.6	87.9	95	95.1		
Previous CABG	9	12	51.3	36.4	19	16.3		
LVEF	—	—	—	—	53 ± 11.5	53.6 ± 11.3		
PAD	11	20	—	—	—	—		
CKD/dialysis	3	8	5.1	0.0	41.8	35.8		
Presentation %								
Stable angina	—	—	—	—	57.9	57.1		
UA	—	—	—	—	29.1	27.7		
NSTEMI	24	29	—	—	8.3	6.0		
STEMI	48	4	—	—	0.1	0		

Abbreviations: CABG, coronary artery bypass grafting; CKD, chronic kidney disease; DM, diabetes mellitus; HLD, hyperlipidemia; HTN, hypertension; LVEF, left ventricular ejection fraction; MI, myocardial infarction; NSTEMI, non‐ST elevation myocardial infarction; OA, orbital atherectomy; PAD, peripheral arterial disease; PCI, percutaneous coronary intervention; RA, rotational atherectomy; STEMI, ST elevation myocardial infarction; UA, unstable angina.

**Table 3:** Study design, methods, exclusion criteria and outcomes of included studies.

Study	Design	Study period	Settings	Follow‐up	Exclusion criteria	Outcomes
Okamoto et al. 2023 [23]	RCT	2019–2021	Single centre	8 months	Patients with ostial left main artery or ostial right coronary artery lesions, female patients with childbearing potential or a life expectancy of fewer than 12 months.	Primary outcome was stent expansion defined as minimum stent area divided by distal reference lumen area. The secondary outcomes were other OCT findings including tissue modification assessed by the OCT images before and right after the atherectomy.
Yasumura et al. 2024 [14]	ROS	2017–2022	Single centre	1 year	Patients presenting with in‐stent restenosis lesions, myocardial infarction with ST‐segment elevation (STEMI), or those in cardiogenic shock.	Primary endpoint was minimum stent area (MSA) post‐PCI. Secondary endpoints included MSA at CN site and 1‐year target vessel failure (TVF), defined as a composite of cardiac death, target‐vessel myocardial infarction or target vessel revascularisation.
Rola et al. 2023 [15]	ROS	2014–2023	Multicentre	30 days	—	Primary endpoint was the occurrence of in‐hospital major adverse cardiac and cerebrovascular events (MACCE). Secondary endpoints included the occurrence of MACCE after 1‐month postprocedure, all kinds of revascularisation procedures, cerebrovascular episodes and stent restenosis.
Barrett et al. 2020 [16]	ROS	2014–2018	Multicentre	30 days	Individuals with missing information regarding lesion severity or procedural technique.	MACE, defined as a composite of all‐cause mortality, MI, TVR and stroke
Lee et al. 2017 [22]	ROS	2012–2016	Single centre	30 days	Patient presenting as STEMI.	Primary end point was the rate of 30‐day MACE defined as the composite of death, MI, TVR and stroke.
Meraj et al. 2018 [21]	POS	2011–2017	Multicentre	In hospital	Any patient below 18 years of age and those treated with laser atherectomy.	Primary end point was MI and safety outcomes included significant dissection, perforation, cardiac tamponade and vascular complications.
Koifman et al. 2018 [20]	ROS	2013–2016	Single centre	—	—	The primary end point was the increase in troponin after the procedure. Secondary end points included procedural success, all‐cause mortality, Q‐wave MI, TVR, TLR and stent thrombosis.
El Hajj et al. 2020 [17]	ROS	2016–2017	Single centre	1 year	Subjects receiving balloon angioplasty, bare metal stents or lacking 1 year follow‐up.	MACE, defined as a composite of all‐cause mortality, MI, TVR and stroke
Megaly et al. 2021 [13]	ROS	2016–2017	Multicentre	30 days	Patients with missing data on in‐hospital mortality, who had combined RA and OA, and whose admission month was December.	The primary outcome was in‐hospital mortality. Secondary outcomes included in‐hospital coronary artery bypass graft surgery (CABG), coronary perforation, ischemic or haemorrhagic stroke, vascular complications, renal complications, discharge to a nursing facility, length of hospital stay, the cost of index hospitalization and nonelective 30‐day readmission.
Chambers et al. 2018 [19]	ROS	2010–2016	Single centre	30 days	In‐stent restenosis.	MACE, defined as a composite of death, MI and TVR at 30 days postprocedural.
Okamoto et al. 2019 [18]	ROS	2013–2016	Single centre	1 year	Patients with cardiogenic shock and missing data for angiographic calcification.	Primary 1‐year clinical outcome was MACE, defined as a composite of death, MI or TLR.

Abbreviations: MACE, major adverse cardiac events; MI, myocardial infarction; OA, orbital atherectomy; POS, prospective observational study; ROS, retrospective observational study; RA, rotational atherectomy; RCT, randomised control trial; STEMI, ST Segment elevation myocardial infarction; TVR, target vessel revascularisation.

### 3.3. Primary Outcomes

#### 3.3.1. MACE

No statistically significant difference was observed in short‐term MACE between RA and OA (OR = 0.93, 95% CI: 0.62–1.39, *p* = 0.73), with low heterogeneity (*I* ^2^ = 0*%*, *p* = 0.90). However, long‐term MACE was significantly higher in the RA group compared to OA (OR = 1.51, 95% CI: 1.01–2.26, *p* = 0.04) (Figure 3a).

#### 3.3.2. All‐Cause Mortality

Short‐term all‐cause mortality showed no statistically significant difference between RA and OA (OR = 1.09, 95% CI: 0.31–3.77, *p* = 0.90), with moderate heterogeneity (*I* ^2^ = 46*%*, *p* = 0.11). Similarly, long‐term all‐cause mortality was not significantly different between groups (*OR* = 3.24, 95% CI: 0.90–11.70, *p* = 0.07), with low heterogeneity (*I* ^2^ = 0*%*, *p* = 0.35) (Figure 3b).

#### 3.3.3. Cardiac Mortality

No significant difference was observed in short‐term cardiac mortality between RA and OA (OR = 0.54, 95% CI: 0.07–4.46, *p* = 0.57), with low heterogeneity (*I* ^2^ = 0*%*, *p* = 0.52). Similarly, long‐term cardiac mortality did not differ significantly between the groups (OR = 2.57, 95% CI: 0.27–24.25, *p* = 0.41) (Figure 4a).

#### 3.3.4. Nonfatal MI

Short‐term nonfatal MI was significantly higher in the RA group compared to OA (OR = 1.77, 95% CI: 1.05–2.98, *p* = 0.03), with low heterogeneity (*I* ^2^ = 0*%*, *p* = 0.72). Long‐term nonfatal MI showed no significant difference (OR = 1.12, 95% CI: 0.73–1.70, *p* = 0.61), with low heterogeneity (*I* ^2^ = 0*%*, *p* = 0.40) (Figure 4b).

#### 3.3.5. TVR

No significant difference was found for short‐term TVR between RA and OA (OR = 0.99, 95% CI: 0.63–1.55, *p* = 0.96; *I* ^2^ = 0*%*, *p* = 0.37). However, long‐term TVR was significantly higher in the RA group (OR = 2.51, 95% CI: 1.12–5.64, *p* = 0.03), with low heterogeneity (*I* ^2^ = 0*%*, *p* = 0.73) (Figure 5a).

### 3.4. Secondary Outcomes

#### 3.4.1. Procedural Complications

The incidence of coronary dissection was significantly lower in the RA group compared to the OA group (OR = 0.38, 95% CI: 0.20–0.72, *p* = 0.003), with no heterogeneity (*I* ^2^ = 0*%*, *p* = 0.53) (Figure 5b). Similarly, cardiac tamponade was not significantly different between groups (OR = 0.55, 95% CI: 0.11–2.88, *p* = 0.48), with low heterogeneity (*I* ^2^ = 8*%*, *p* = 0.38) (Figure 5c). Device‐induced coronary perforation showed no significant difference between RA and OA (OR = 0.50, 95% CI: 0.17–1.44, *p* = 0.20), with low heterogeneity (*I* ^2^ = 32*%*, *p* = 0.21) (Figure 6a). For slow‐flow/no‐reflow, there was no significant difference between RA and OA (OR = 1.31, 95% CI: 0.60–2.87, *p* = 0.49; *I* ^2^ = 0*%*, *p* = 0.97) (Figure 6b).

#### 3.4.2. Procedural Characteristics

No significant difference was found in fluoroscopy time between RA and OA (MD = 3.59 min, 95% CI: −0.37 to 7.55, *p* = 0.08; *I* ^2^ = 91*%*) (Figure 6c). Similarly, contrast volume did not differ significantly between groups (MD = −3.13 mL, 95% CI: −18.44 to 12.19, *p* = 0.69; *I* ^2^ = 86*%*) (Figure 6d). Both outcomes demonstrated substantial heterogeneity, indicating variability among included studies. Subgroup analysis stratified by clinical presentation or procedural factors was considered; however, these were not feasible due to insufficient reporting in the included studies.

### 3.5. Sensitivity Analysis

A sensitivity analysis was performed to assess the influence of Megaly et al., which contributed a disproportionately large sample size compared with the other included studies. In the primary analysis excluding Megaly et al., short‐term and long‐term all‐cause mortality were not significantly different between RA and OA. When Megaly et al. was included, the direction of effect for most outcomes remained consistent; however, mortality estimates changed materially [13]. Short‐term all‐cause mortality became significantly higher in the RA group (OR = 1.61, 95% CI: 1.38–1.87, *p* < 0.00001; *I* ^2^ = 52*%*), and long‐term all‐cause mortality was also significantly higher in the RA group (OR = 3.98, 95% CI: 1.13–14.02, *p* = 0.03). Long‐term MACE and long‐term TVR remained significantly higher in the RA group, whereas short‐term MACE, cardiac mortality, long‐term nonfatal MI, short‐term TVR, device‐induced perforation and slow‐flow/no‐reflow remained nonsignificant. These findings indicate that mortality outcomes were particularly sensitive to the inclusion of this large observational dataset, whereas most nonmortality outcomes were directionally stable.

### 3.6. Methodology and Risk of Bias

The quality of the RCTs was assessed using the Cochrane Collaboration Risk of Bias tool (RoB 2), and the results are summarised in Figure 1. The included randomised study demonstrated low risk of bias across most domains, with some concerns noted in the domain relating to missing outcome data, resulting in an overall judgement of some concerns. The retrospective observational studies were evaluated using the NOS, as summarised in Table 1. According to the AHRQ standards, eight studies were classified as good quality, whereas two studies were rated as poor quality. No studies were categorised as fair quality.

## 4. Discussion

This updated systematic review and meta‐analysis synthesised evidence from 11 studies involving more than 80,000 patients and provides a contemporary comparison of rotational and OA for severely calcified coronary lesions. The analysis incorporated a broad range of clinically relevant outcomes, including MACE, mortality, MI, repeat revascularisation, procedural complications, fluoroscopy exposure, and contrast use.

The pooled findings do not establish uniform clinical superiority of either atherectomy strategy. Short‐term MACE and cardiac mortality were similar between RA and OA. Nevertheless, patients treated with RA had higher pooled rates of short‐term nonfatal MI, long‐term MACE and long‐term TVR. All‐cause mortality did not differ significantly between the two approaches at either short‐ or long‐term follow‐up in the primary analysis. Fluoroscopy duration and contrast use were also not significantly different, although substantial heterogeneity was observed for both procedural measures.

The procedural safety findings demonstrated a different pattern. Coronary dissection occurred less frequently in the RA group, whereas no significant differences were detected for cardiac tamponade, device‐induced coronary perforation or slow‐flow/no‐reflow. These results therefore suggest that potential advantages and disadvantages differ according to the individual endpoint considered, rather than favouring one atherectomy device consistently across all measures of procedural safety and clinical effectiveness.

Several factors may contribute to the higher pooled long‐term MACE and TVR rates observed following RA. The two technologies interact with calcified plaque through different mechanical principles. RA advances a concentrically rotating burr through the lesion, whereas OA uses an eccentrically positioned crown whose orbital movement modifies calcium circumferentially [9]. The latter mechanism may permit broader calcium modification and potentially facilitate more complete stent expansion, which could theoretically influence subsequent restenosis and repeat revascularisation [24–26]. Similarly, the higher short‐term incidence of nonfatal MI associated with RA could potentially relate to differences in particulate embolisation or transient impairment of the distal microcirculation. Such explanations remain speculative, however, and cannot be established from the aggregate data available in this review.

The lower rate of coronary dissection observed with RA also requires cautious interpretation. OA produces an expanding orbital trajectory rather than the predominantly concentric forward movement characteristic of RA [27]. In vessels with eccentric calcium, tortuosity, or substantial guidewire bias, this movement could conceivably create different lateral forces at the boundary between rigid calcific plaque and the more compliant arterial wall [27]. This provides one possible mechanistic explanation for the observed difference in dissection, although procedural selection may be equally important. Operators may preferentially use OA in particular anatomical or lesion subsets, including more complex cases treated at specialist centres [24–26]. Because lesion morphology, intravascular imaging characteristics and detailed procedural techniques were incompletely reported across the included studies, the explanation for this association cannot be determined with confidence. The finding should therefore be regarded as hypothesis‐generating.

The sensitivity analysis also demonstrated that estimates of mortality were particularly dependent on the contribution of individual large observational datasets. When Megaly et al. was excluded from the primary analysis, neither short‐ nor long‐term all‐cause mortality differed significantly between RA and OA. Reintroduction of this study resulted in statistically significant increases in both short‐ and long‐term mortality among patients treated with RA [13]. This change illustrates how a very large observational cohort can dominate pooled estimates when the remaining evidence base is considerably smaller. In contrast, the direction of most nonmortality outcomes was maintained across the sensitivity analyses. The available evidence therefore does not support a definitive conclusion regarding a survival advantage for either device.

The present findings also extend previous pooled evidence. Khan et al. [7] reported in 2020 that overall MACE, mortality and TVR did not differ significantly between RA and OA, although OA was associated with lower long‐term MACE and short‐term MI. The current analysis incorporates a substantially larger patient population and additional contemporary evidence. It similarly identifies higher long‐term MACE, long‐term TVR and short‐term nonfatal MI with RA. However, the instability of the mortality estimates in sensitivity analysis reinforces the need to distinguish statistical associations derived from observational datasets from evidence of a true treatment effect.

An additional challenge is that severe CAC represents a heterogeneous group of coronary lesions rather than a single anatomical phenotype. Calcification may be concentric or eccentric and may include calcified nodules, long calcified segments or lesions associated with chronic total occlusion [28, 29]. Contemporary device selection is therefore often driven by lesion‐level anatomy and increasingly by intravascular imaging. In particularly complex lesions, atherectomy may also form only one component of a broader calcium‐modification strategy involving more than one technology.

Consequently, treating RA and OA as completely interchangeable and mutually exclusive approaches may not fully reflect contemporary PCI practice. Apparent outcome differences may partly arise from confounding by indication, whereby operators select a particular technology because of lesion morphology, anatomical complexity or perceived procedural risk. The available studies inconsistently reported calcium arc, lesion length, calcified nodules, chronic total occlusion status, imaging guidance and combined plaque‐modification techniques, preventing meaningful subgroup analysis according to these characteristics.

This limitation is increasingly relevant as coronary intervention moves towards more detailed plaque and lesion phenotyping. Modern invasive and noninvasive imaging can provide information extending beyond simple angiographic stenosis severity, allowing assessment of calcification pattern and other features that may influence procedural strategy [29, 30]. As imaging‐guided PCI becomes more widely adopted, selection of an atherectomy device may increasingly depend on specific anatomical characteristics rather than a uniform device preference [30]. Without consistent lesion‐level imaging data in the published comparative literature, it remains difficult to determine whether the associations identified in this meta‐analysis arise from intrinsic differences between the devices or from differences in the patients and lesions for which they were selected.

Geographical and institutional differences in device use should also be considered. RA has been adopted widely across multiple healthcare systems, whereas OA has historically been concentrated in selected centres and has been used particularly extensively in the United States. Outcomes observed with OA may therefore partly reflect treatment by operators and centres with specific expertise in the technology. Such centre‐ and operator‐level factors create an additional potential source of selection bias and may reduce the applicability of pooled findings to institutions with lower atherectomy volumes or limited experience with OA.

This review has several strengths. It incorporates an updated evidence base comprising 11 studies and more than 80,000 patients and evaluates multiple clinical and procedural outcomes. Separating short‐ and long‐term endpoints provides greater temporal detail than analyses combining outcomes across markedly different follow‐up periods. The use of sensitivity analyses also permits assessment of how strongly individual large datasets influence the overall conclusions.

Several limitations must nevertheless be considered. Most of the available evidence was observational, leaving the analysis susceptible to residual confounding, treatment‐selection bias and confounding by indication. Differences between studies in patient characteristics, lesion severity, operator experience, duration of follow‐up, intravascular imaging use and adjunctive plaque‐modification strategies may also have influenced the pooled estimates. In particular, inconsistent reporting of intravascular imaging is important because imaging guidance may influence lesion preparation and stent optimisation in heavily calcified PCI [24]. Mortality estimates were additionally sensitive to the inclusion of a single large observational study, demonstrating the limited robustness of this endpoint. Finally, the number of studies contributing to most outcomes was below the threshold required for reliable formal assessment of funnel‐plot asymmetry; publication bias therefore could not be adequately evaluated for these endpoints.

Further evidence should ideally come from adequately powered, multicentre, head‐to‐head randomised trials comparing RA and OA within contemporary imaging‐guided PCI practice. Future studies should report detailed lesion phenotypes, intravascular imaging findings, operator and institutional experience and the use of hybrid calcium‐modification approaches. Prespecified analyses of clinically important subgroups, including patients with chronic kidney disease, left main coronary disease and multivessel disease, would help determine whether populations derive greater benefit from one approach. Evaluation of comparative cost‐effectiveness would also be valuable when considering broader implementation of these technologies.

## 5. Conclusion

In this updated meta‐analysis involving more than 80,000 patients, OA was associated with lower pooled rates of long‐term MACE, short‐term nonfatal MI and long‐term TVR, whereas RA was associated with a lower incidence of coronary dissection. The primary analysis did not demonstrate a consistent difference in all‐cause mortality, and mortality estimates changed according to the inclusion of a large observational cohort. Because the available literature remains predominantly observational and is susceptible to residual confounding, treatment‐selection bias, differences in lesion complexity, operator experience, intravascular imaging use and adjunctive procedural strategies, these findings cannot establish causal superiority of either device. Selection between RA and OA should therefore remain tailored to lesion characteristics, imaging findings and operator expertise. Adequately powered, imaging‐guided randomised trials directly comparing the two approaches are needed to determine whether the observed differences in clinical and revascularisation outcomes translate into meaningful long‐term benefit.

## Author Contributions

Eyad Jamileh collected and analysed the data, contributed to the study design, drafted the original manuscript and revised the manuscript. Ahmed Elmewafy collected and analysed the data, contributed to drafting the manuscript and revised the manuscript. Fahad Muhammad critically revised the manuscript. Ahmed Ali Kayyale contributed to drafting the manuscript. Usama Al Bushe Ade collected the data. Abdelmadjid Zinet collected the data. Umar Faruq Ali contributed to drafting the manuscript. Ibrahim Antoun conceived and designed the study, critically revised the manuscript, approved the final version and accepts responsibility for the integrity of the work as a whole. Eyad Jamileh and Ahmed Elmewafy contributed equally as joint first authors.

## Funding

No funding was received for this manuscript.

## Ethics Statement

A statement of ethics is not applicable because this study is based exclusively on published literature.

## Conflicts of Interest

The authors have no conflicts of interest to declare.

## Supporting Information

Additional supporting information can be found online in the Supporting Information section.

## Supporting information


**Supporting Information 1** File S1: PRISMA 2020 Checklist. This file outlines adherence to the PRISMA 2020 reporting guidelines, detailing where each checklist item is addressed within the manuscript.

**Supporting Information 2** File S2: Search Strategy. This file provides the complete electronic search strategies used across all databases, including keywords, Boolean operators and filters applied to identify eligible studies.

## Data Availability

The data that support the findings of this study are available from the corresponding author upon reasonable request.
