# Supplementary material for: Comparative Outcomes of Rotational Versus Orbital Atherectomy in Calcified Coronary Lesions: An Updated Systematic Review and Meta‐Analysis
Source: Cardiovasc Ther. 2026 Sep 2;2026:4716803. doi: 10.1155/cdr/4716803 (PMC13536482; doi:10.1155/cdr/4716803)
Supplement: Supplementary file 2 [file CDR-2026-4716803-s002.docx]

# Search Strategy

## 1. CENTRAL (Cochrane Central Register of Controlled Trials)

(
("rotational atherectomy" OR rotablator OR "rotablation")
OR
("orbital atherectomy" OR diamondback OR "orbital atherectomy system")
)
AND
(
"percutaneous coronary intervention"
OR PCI
OR angioplasty
OR "coronary intervention"
OR "coronary revascularization"
)
AND
(
coronary
OR "coronary artery"
OR "coronary lesion"
OR "calcified lesion"
OR calcification
OR "coronary artery calcification"
OR "coronary calcification"
OR "plaque modification"
OR "left main"
OR CAD
)
NOT
(
"peripheral artery"
OR femoral
OR carotid
OR renal
)

## 2. EMBASE (Ovid)

1. 'rotational atherectomy'/exp OR 'rotational atherectomy':ti,ab OR rotablator:ti,ab OR rotablation:ti,ab

2. 'orbital atherectomy'/exp OR 'orbital atherectomy':ti,ab OR diamondback:ti,ab OR 'orbital atherectomy system':ti,ab

3. 1 OR 2

4. 'percutaneous coronary intervention'/exp OR 'percutaneous coronary intervention':ti,ab OR pci:ti,ab OR angioplasty:ti,ab OR 'coronary intervention':ti,ab OR 'coronary revascularization':ti,ab

5. coronary:ti,ab
OR 'coronary artery':ti,ab
OR 'coronary lesion':ti,ab
OR 'calcified lesion':ti,ab
OR 'coronary artery calcification':ti,ab
OR 'coronary calcification':ti,ab
OR calcification:ti,ab
OR 'plaque modification':ti,ab
OR 'left main':ti,ab
OR cad:ti,ab

6. 3 AND 4 AND 5

7. 'peripheral artery':ti,ab OR femoral:ti,ab OR carotid:ti,ab OR renal:ti,ab

8. 6 NOT 7

## 3. PubMed

(
("rotational atherectomy"[Title/Abstract]
OR rotablator[Title/Abstract]
OR rotablation[Title/Abstract])

OR

("orbital atherectomy"[Title/Abstract]
OR diamondback[Title/Abstract]
OR "orbital atherectomy system"[Title/Abstract])
)

AND

(
"percutaneous coronary intervention"[Title/Abstract]
OR PCI[Title/Abstract]
OR angioplasty[Title/Abstract]
OR "coronary intervention"[Title/Abstract]
OR "coronary revascularization"[Title/Abstract]
)

AND

(
coronary[Title/Abstract]
OR "coronary artery"[Title/Abstract]
OR "coronary lesion"[Title/Abstract]
OR "calcified lesion"[Title/Abstract]
OR calcification[Title/Abstract]
OR "coronary artery calcification"[Title/Abstract]
OR "coronary calcification"[Title/Abstract]
OR "plaque modification"[Title/Abstract]
OR "left main"[Title/Abstract]
OR CAD[Title/Abstract]
)

NOT

(
"peripheral artery"[Title/Abstract]
OR femoral[Title/Abstract]
OR carotid[Title/Abstract]
OR renal[Title/Abstract]
)
